# Is intra-articular injection of autologous micro-fragmented adipose tissue effective in hip osteoarthritis? A three year follow-up

**DOI:** 10.1007/s00264-022-05611-x

**Published:** 2022-10-28

**Authors:** Simone Natali, Daniele Screpis, Michele Romeo, Stefano Magnanelli, Giuseppe Rovere, Amarossi Andrea, Lawrence Camarda, Claudio Zorzi

**Affiliations:** 1 Department of Orthopaedics, IRCCS Ospedale Sacro Cuore Don Calabria, Negrar di Valpolicella, Italy; 2 Department of Orthopaedic Surgery (DICHIRONS), University of Palermo, Palermo, Italy; 3 Department of Orthopaedics and Traumatology, Fondazione Policlinico Universitario A. Gemelli IRCCS—Università Cattolica del Sacro Cuore, Rome, Italy

**Keywords:** Hip osteoarthritis, Autologous micro-fragmented adipose tissue, Adipose-derived mesenchymal stem cells

## Abstract

**Background:**

Recently, increased attention on regenerative medicine and biological injective treatments have been proposed to restore native cartilage. Micro-fragmented adipose tissue (MFAT) has been studied for its anti-inflammatory, paracrine, and immunomodulatory effects. The long-term effects of MFAT are still poorly understood: the aim of the present study is to demonstrate how hip articular injections with autologous MFAT can have an impact on clinical outcomes.

**Methods:**

Seventy-one consecutive patients affected by early hip osteoarthritis underwent an ultrasound-guided hip injection of autologous MFAT between June 2017 and December 2018. Patients were divided into four groups according to the Oxford Hip Score. All patients received 4 mL of autologous micro-fragmented adipose tissue under an ultrasound guide. A clinical evaluation was done between 29 and 41 months after the initial treatment. During this follow-up period, we recorded any new treatment the patients had done, whether that be injection or arthroplasty surgery.

**Results:**

The study included 55 patients. Out of 55 patients, 28 saw benefits and were in no need of further treatment. Moreover, the score between the beginning and control increased by 6.9 points. Ten patients underwent a new articular injection: the mean time between the two injections was 635.7 ± 180 days. Seventeen patients underwent total hip replacement: the mean period between the autologous MFAT injection and the surgery was 495 days.

**Conclusion:**

This study found that intra-articular injections with autologous MFAT achieve beneficial clinical results in patients affected by early to moderate hip osteoarthritis, with an OHS between 48 and 30. Furthermore, these subjects are the ideal patients for whom this treatment obtains good clinical results.

## Introduction

Osteoarthritis (OA) is a joint disease caused by a degeneration of the cartilage and underlying bone. The most common symptoms are pain and stiffness located in the affected joint. It is estimated that 250 million people worldwide are affected by OA, and it is one of the most common causes of activity loss in adults [1, 2]. A systematic analysis of the Global Burden of Disease Study (2017) calculated that the global age-standardized years lived with disease (YLD) rate in 2017 increased by 9.6% compared to 1990 [3].

Non-invasive solutions, such as exercise, physical therapies, oral nonsteroidal anti-inflammatory drugs (NSAIDs), and injective treatments, have been suggested for the management of early osteoarthritis [4–7]. Recently, increased attention has been observed on regenerative medicine with the use of biological injective treatments to restore the native cartilage. Mesenchymal stem cells (MSCs) are multipotent adult stem cells that are present in multiple tissues, and they have been studied for anti-inflammatory, paracrine, and immunomodulatory effects [8].

The sources of MSCs include the bone marrow, adipose tissue, peripheral blood, muscle, amniotic fluid, umbilical cord, and placenta [9]. Cells isolated from the adipose tissue and bone marrow exhibit typical MSC characteristics: this is confirmed by the expression of a typical set of surface proteins and specific stem cell markers (CD90, CD73, CD105, and CD44) which are shared by adipose-derived mesenchymal stem cells (ADMSCs) and bone marrow-derived stem cells (BMSCs) [10].

Several adipose tissue processing methods have been developed for ADMSCs. These can be divided into two main groups: enzymatic and non-enzymatic processing techniques [7]. In the first group, enzymes such as collagenase, trypsin, and dispase are used to obtain the complete digestion of the lipoaspirate. Non-enzymatic isolation methods are based on centrifugation, pressure, filtration, and washing.

Micro-fragmented adipose tissue (MFAT) contains the stromal vascular fraction (SVF), which could be considered a niche for ADMSCs. SVF includes a high concentration of stem cells, growth factors, and release factors such as cytokines and chemokines, inducing the healing of the host cells [11].

In consideration of the potential this type of treatment has for OA, its relative usefulness and safety have been well-documented in several short-term studies. However, its effect in the long term is still poorly investigated: the present study aims to demonstrate how hip articular injections with autologous MFAT can have an impact on clinical outcomes.

## Material and methods

This study has been approved by the Ethics Committee and confirms the declaration of Helsinki and its modifications. Seventy-one consecutive patients affected by hip osteoarthritis underwent an ultrasound-guided hip injection of MFAT between June 2017 and December 2018 in IRCCS Sacro Cuore—Don Calabria Hospital (Negrar di Valpolicella, VR, Italy).

The inclusion criteria were hip pain resistance to NSAIDs in the past 4 months or more, functional limitations, and/or the failure of previous conservative treatments. Patients with a history of trauma at the symptomatic hip were excluded from the study.

Lipogems® ortho kit (Lipogems International SpA, Milan, Italy) is a kit able to obtain a final product of MFAT. All patients were injected with 4 mL of autologous MFAT in the joint under ultrasound guidance. The post-treatment protocol consisted in using crutches for three days, antithrombotic prophylaxis, and application of an elastic waistband for 15 days at the harvesting site.

Before this procedure, a questionnaire with the Oxford Hip Score was administered to enrolled patients. The Oxford Hip Score (OHS) is a joint-specific outcome measurement tool designed to assess disability in patients undergoing total hip replacement. The OHS is also used to assess patients after alternative non-surgical interventions. Subjects were divided into four groups according to the Oxford Hip score: patients with early (E), moderate (M), moderate to severe (MS), and severe (S) osteoarthritis. Pre-operative radiological evaluation was obtained and the grade of osteoarthritis was classified according to Kellgren Lawrence (KL) classification [12], based on standard X-rays of the affected hip. The visual analog scale (VAS) was used to record pre-operative pain intensities.

A clinical evaluation was performed at follow-up, including VAS evaluation and radiological evaluation based on new radiographs. If the patient underwent another injection or surgery procedure, the period between autologous MFAT injection and the following treatment was recorded. There were three groups: those who had not received any other treatment, those who had received no joint infiltration, and those who had undergone a prior hip replacement surgery.

## Results

Seventy-one patients were contacted in April 2021: 16 patients did not complete the final follow-up, and 55 patients joined the study. There were 22 males and 33 females with a mean age of 52.5. Furthermore, there were 34 right hips and 21 left hips examined. The mean pre-operative BMI was 23.7 ± 3.2. The mean follow-up was 35 ± six months (Table 1). Only one adverse event in the follow-up was recorded: some days after the procedure, the presence of a deep bruise near the harvest site was found, which was resolved over the course of a few months.

**Table 1:** Background data of the population

Background data of the population	
Number of patient	71
Patient enrolled in the study	55
Patient lost in the follow up	16
Sex	Male: 22
	Female: 33
Age (y/o)	Mean: 52.5
	SD: ± 10.9
BMI	Mean: 23.72
Side	SD: ± 3. 2 Right: 34 hips Left: 21 hips
Follow-up (months)	Mean: 35
	SD: ± 6

The mean pre-operative grade of osteoarthritis according to Kellgren—Lawrence classification was 1.9 ± 0.8, and the pre-operative pain evaluation according to the VAS scale was 4.7 ± 1.1. According to the OHS, 22 patients were categorized as early OA, 12 patients as moderate OA, 16 patients as moderate to severe OA, and six patients as severe OA. Clinical results and further treatments evaluated are detailed in Table 2.

**Table 2:** Groups of population and treatments during the follow-up

	*N* (total)	Subgroups
Early osteoarthritis (40–48)—E	21	No other treatments: 15 New articular injection: 5 Total hip replacement: 1
Moderate osteoarthritis (30–39)—M	12	No other treatments: 6 New articular injection: 4 Total hip replacement: 2
Moderate to severe osteoarthritis (20–29)—MS	16	No other treatments: 7 New articular injection:/ Total hip replacement: 9
Severe osteoarthritis (0–19)—S	6	No other treatments:/ New articular injection: 1 Total hip replacement: 5

There were not any recorded improvements observed in the radiological follow-up; there were eight recorded worsening cases (from grade 2 to grade 3). Although none of the patients worsened in terms of pain evaluation when compared to the follow-up and their respective pre-operative conditions as the new mean VAS score was 1.8 ± 0.4.

Twenty-eight patients (15 E, 6 M, 7, MS) did not undergo any new articular injections (corticosteroid, viscosupplementaion, platelet-rich plasma, MFAT) or total hip replacement (Table 3). The mean age of this group was 51.5 ± 11, and the mean BMI was 22.5 ± 3.12. The mean improvement of the OHS was 6.9 ± 5.6 points.

**Table 3:** Analysis of the groups

No other treatments		
*N* total	28	
Age (y/o)	51.5±11	
Δ OHS	6.9±5.6	
	N	Δ OHS
E (40–48)	15	3.33±2.55
M (30–39)	6	12.5±2.5
MS (20–29)	7	9.6±8.2
New injection		
*N* total	10	
Age (y/o)	52±6.17	
Δ New injection – MFAT (days)	635.7±180	
	*N*	Δ New injection – MFAT (days)
E (40–48)	5	494±106.7
M (30–39)	4	815.5±50.9
MS (20–29)	0	/
S (0–19)	1	424
Total hip replacement		
N total	17	
Age (y/o)	55.12±5.7	
BMI	24.6±3.2	
Δ Surgery – MFAT (days)	495±222	
	*n*	Δ Surgery – MFAT (days)
E (40–48)	1	453
M (30–39)	2	777±151
MS (20–29)	9	503±220
S (0–19)	5	375±191

For those who did not receive any additional treatment, the mean improvement in group E (early OA) was 3.33 ± 2.55 points, 12.5 ± 2.5 for group M (moderate OA), and 9.6 ± 8.2 for the MS group (moderate to severe OA).

Ten patients underwent a new articular injection (5 E, 4 M, 1 S) with an overall basal OHS of 37 ± 10.7 (Table 3). The mean age was 52 ± 6.17. The mean time between the two injections was 635.7 ± 180 days: 494 ± 106.7 days in group E (early OA), 815.5 ± 50.9 days in group M (moderate OA), and 424 days in group MS (moderate to severe OA). Improvements in OHS in group E were 1.2 ± 1.09, 7.25 ± 4.85 for group M, and 13 for group S. The overall improvement was 4.8 ± 5.02.

Seventeen patients underwent total hip replacement (1 E, 2 M, 9 MS, 5 S) (Table 3). This group of patients was composed of nine males and eight females. The mean age was 55.12 ± 5.7, and the mean BMI was 24.6 ± 3.2. The mean time between the autologous MFAT injection and the time of the surgery was of 495 ± 222 days: 453 days for group E, 777 ± 151 days for group M, 503 ± 220 for group MS, and 375 ± 191 for group S. Any changes between the time of articular injection and the time of the surgery were recorded.

## Discussion

The primary result that the present study highlighted is the efficacy and the safety of autologous MFAT in the management of early and moderate hip OA in the long term while also improving the clinical condition of the subjects studied with an overall reduction in pain. Patients that underwent other treatments, such as new articular injections or total hip replacement, did not perceive a worsening of their clinical condition after the treatment compared to the pre-operative period. In addition, no adverse events were observed in patients at the follow-up time, thus demonstrating the safety profile of this procedure. These data are in accordance with those present in the current literature. For example, Lee et al. [13] found that intra-articular injection of autologous ADMSCs in the knee provides improvements in pain relief and satisfactory function when compared to a placebo in a six month follow-up.

Studies have demonstrated that the cells present in tissue collected by the Lipogems® system have the ability to differentiate into osteocyte, vascular, and chondrocyte cells [14]. The injection of adipose tissue has three main ways of reducing pain: anti-inflammatory, mechanical, and biological. It has been demonstrated that ADMSCs release cytokines [15], and the adipose tissue injection has a lubricating capacity [16]. Furthermore, the capacity to increase the glycosaminoglycans (GAG) content in hyaline cartilage supports the biological action in reducing pain [17]: the GAG content in the cartilage was analyzed using functional MRI through delayed gadolinium (Gd)-enhanced magnetic resonance imaging (dGEMRIC). It was demonstrated that a single articular injection of autologous ADMSCs improves the GAG content, thus restoring the cartilage. Furthermore, Koh et al. study [18] found that the cartilaginous MRI score improved after MFAT injection into the knee; clinical improvements were contingent on the number of stem cells that were injected. These three ways explain how it achieved clinical effects in the short, middle, and long-term periods.

Our study has demonstrated that 28 patients treated with autologous MFAT injection did not receive any new treatment in the follow-up period. By dividing the patients, according to the OHS, the major relief of this treatment was achieved in patients with “early” and “moderate” OA; the mean improvement was 3.33 ± 2.55 and 12.5 ± 2.5 points, respectively. Minor improvements were observed in the group with “early” OA, while the “moderate” OA group was more symptomatic, thus achieved more of a benefit from the treatment, and they improved their overall OHS.

The remaining patients can be divided into two groups: the first group includes subjects that underwent a total hip replacement, while the second group includes those that got a new articular injection (either corticosteroid, viscosupplementaion, platelet-rich plasma, or MFAT).

The total hip replacement group was composed mainly of patients, in accordance with the OHS, affected by “moderate to severe” and “severe” OA. They did not report any change in their clinical conditions between the time of the articular injection and the time of the surgery. Kraeutler et al. [19] found a mean time of 1.3 years between the first injection of low molecular weight hyaluronic acid and surgery (total hip replacement or hip resurfacing). In the same study, those who received platelet-rich plasma were found to have a mean time of 0.73 years. In our study, the mean time was 1.36 years (495 days).

The group of patients treated with a new articular injection was mainly composed of patients, in accordance with the OHS, affected by “early” and “moderate” OA. A clinical improvement was demonstrated in the period between the two treatments: 494 ± 106.7 days in group E (early OA), 815.5 ± 50.9 days in group M (moderate OA), and 424 days in group MS (moderate to severe OA).

Group MS is the borderline: in this group, seven patients did not require any further treatment at the follow-up, while nine patients underwent a total hip replacement. In this group, the indication of autologous MFAT treatment is not clear: it may be considered as a trial to delay a prospective surgery.

Few papers have studied the application of MFAT in hip pathology. Dell’Oca et al. [16] have found a positive outcome in VAS scores at six month follow-up.

The radiological follow-up of this study demonstrates that the treatment discussed is not a disease-modifying treatment; in fact, none of the patients improved their radiological condition. However, we could state that it is a treatment that can help reduce the pain in patients and improve the overall clinical condition, and an improvement in terms of VAS has been demonstrated (4.7 ± 1,1 vs 1.8 ± 0.4).

Studies with a follow-up of 24 or 36 months were conducted on the knee [20] that demonstrated the efficacy of this treatment. A recent study [21] has found a clinical improvement, up to 12 months, in the treatment of ankle OA. When associated with an open or arthroscopic surgery, superior results of autologous MFAT were demonstrated against an isolated surgical approach in the treatment of knee pathology [22].

This study has limitations that need to be acknowledged: the number of patients is limited, and the absence of a control group does not allow us to have an absolute conclusion.

This study demonstrates the efficacy and the safety of autologous MFAT: in early and moderate OA; according to the OHS, intra-articular injections with autologous MFAT reach beneficial clinical results in the long term. Subjects with early and mild OA, with an OHS between 48 and 30, are the ideal patients to obtain a good clinical result. Otherwise, in patients affected by moderate to severe OA, this treatment could be tried to delay surgical procedures. Furthermore, other studies are necessary to evaluate the efficacy of this treatment, with a larger sample size and an extended follow-up period.

## Data Availability

Not applicable.
